# Medulloblastoma-associated mutations in the DEAD-box RNA helicase *DDX3X/DED1* cause specific defects in translation

**DOI:** 10.1016/j.jbc.2021.100296

**Published:** 2021-01-16

**Authors:** Nicolette P. Brown, Ashley M. Vergara, Alisha B. Whelan, Paolo Guerra, Timothy A. Bolger

**Affiliations:** Department of Molecular and Cellular Biology, University of Arizona, Tucson, Arizona, USA

**Keywords:** RNA helicase, medulloblastoma, translation regulation, *Saccharomyces cerevisiae*, stress granule, cancer, translation initiation, DEAD-box protein, start site scanning, M/P, monosome-to-polyribosome, PIC, preinitiation complex, SG, stress granule, UTR, untranslated region

## Abstract

Medulloblastoma is the most common pediatric brain cancer, and sequencing studies identified frequent mutations in *DDX3X*, a DEAD-box RNA helicase primarily implicated in translation. Forty-two different sites were identified, suggesting that the functional effects of the mutations are complex. To investigate how these mutations are affecting DDX3X cellular function, we constructed a full set of equivalent mutant alleles in *DED1*, the *Saccharomyces cerevisiae* ortholog of *DDX3X*, and characterized their effects *in vivo* and *in vitro*. Most of the medulloblastoma-associated mutants in *DDX3X/DED1* (*ded1-mam*) showed substantial growth defects, indicating that functional effects are conserved in yeast. Further, while translation was affected in some mutants, translation defects affecting bulk mRNA were neither consistent nor correlated with the growth phenotypes. Likewise, increased formation of stress granules in *ded1-mam* mutants was common but did not correspond to the severity of the mutants’ growth defects. In contrast, defects in translating mRNAs containing secondary structure in their 5’ untranslated regions (UTRs) were found in almost all *ded1-mam* mutants and correlated well with growth phenotypes. We thus conclude that these specific translation defects, rather than generalized effects on translation, are responsible for the observed cellular phenotypes and likely contribute to *DDX3X*-mutant medulloblastoma. Examination of ATPase activity and RNA binding of recombinant mutant proteins also did not reveal a consistent defect, indicating that the translation defects are derived from multiple enzymatic deficiencies. This work suggests that future studies into medulloblastoma pathology should focus on this specific translation defect, while taking into account the wide spectrum of *DDX3X* mutations.

Cancers of the central nervous system are the second-most prevalent in children (after leukemias), and medulloblastoma is the most common pediatric brain cancer (1). Medulloblastoma is considered an embryonal tumor that originates in the posterior fossa, although it also occurs in adults in some subtypes (2). It has been divided into four subtypes (Wnt, Shh, group 3, and group 4), primarily on the basis of transcriptome differences (3). Medulloblastoma has a 5-year survival rate of 65 to 80% (1, 4), and many survivors suffer long-term developmental and cognitive damage from current therapies (5, 6, 7). Therefore, further research into the molecular defects of medulloblastoma is needed in order to design more effective and targeted treatments that address these shortcomings.

Medulloblastoma was one of the first cancers to be characterized using next-generation sequencing techniques in a series of exome- and genome-wide studies with a combined cohort of several hundred patients (8, 9, 10, 11). One of the most prominent findings from these studies was frequent mutations in the RNA helicase *DDX3X*, which had not previously been linked to medulloblastoma. In fact, in a meta-analysis, *DDX3X* was the second-most commonly mutated gene in medulloblastoma after the transcription factor β-catenin (*CTNNB1*), and the mutations were not limited to a particular subtype, although they were especially frequent in Wnt and adult Shh subtypes (1, 9). Interestingly, the identified mutations were all single-nucleotide variants or small, in-frame indels (see Table 1), suggesting that they are not simply inactivating the gene but have a more specific effect on DDX3X function. On the other hand, the mutations were spread throughout the central “helicase domains” of *DDX3X*, thus complicating any prediction of their effects (Fig. 1*A*).

**Table 1:** Growth phenotypes of *DDX3X/DED1* medulloblastoma-associated mutations

The sites of 42 *DDX3X* point mutations identified in four genome-wide sequencing studies of medulloblastoma patients are organized by encoded amino acid sequence, with the orthologous change in Ded1 sequence also listed. Growth assays of the respective *ded1-mam* mutants were performed at 16, 25, 30, and 37 °C, demonstrating a variety of growth phenotypes: no growth or greatly inhibited growth (−, *red*), moderately inhibited growth (−/+, *pink*), and growth similar to wild-type *DED1* (+, *white*). Lethal *ded1-mam* alleles (those unable to grow at any temperature) are marked in *red* type.

**Figure 1:**
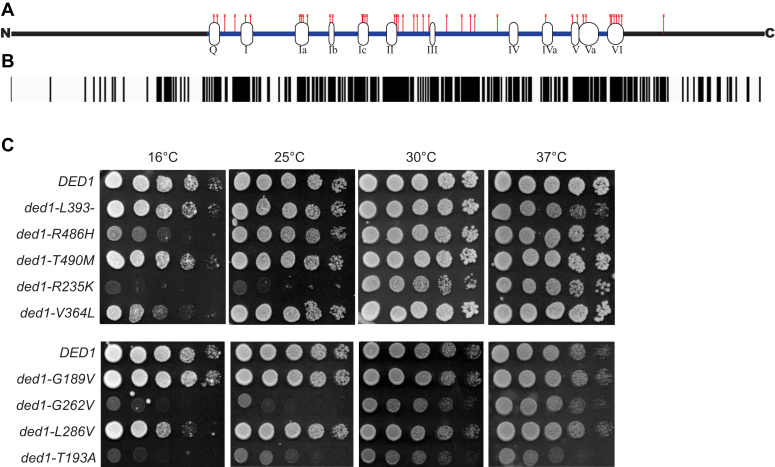
**Medulloblastoma-associated mutations in *DDX3X/DED1* alter growth.***A*, sequence schematic of Ded1/DDX3X with medulloblastoma-associated mutations (*red lines*) and conserved DEAD-box helicase motifs (labeled bubbles). *B*, conservation map of human DDX3X and yeast Ded1. *Black boxes* represent regions of sequence identity. *C*, wild-type *DED1* cells and the indicated *ded1-mam* mutants were grown on nutrient-rich agar at 16, 25, 30, and 37 °C. Representative 5× serial dilutions of cells are shown.

DDX3X and its orthologs, including Ded1 in *Saccharomyces cerevisiae*, are members of the DEAD-box family of RNA helicases, which have critical roles in many facets of RNA biology and gene expression (for review, see (12)). While DDX3X/Ded1 has been implicated in a number of processes, it has been best characterized as a translation factor (13, 14). Current models of eukaryotic translation initiation propose that translation factors, including the 40S ribosomal subunit, first assemble on the mRNA, and this preinitiation complex (PIC) then scans the mRNA, starting from the 5’ cap, for the start codon (15). Once the start codon is recognized, conformational rearrangements commit the PIC to initiation at that site, the 60S subunit joins, and translation elongation can begin. During steady-state conditions, both Ded1 and DDX3X have been shown to stimulate initiation (13, 16, 17, 18). Specifically, both orthologs are proposed to unwind RNA secondary structure in the 5’UTR in order to facilitate start site scanning by the PIC (13, 18, 19). Results from both reporter assays and ribosome profiling (Ribo-seq) studies are consistent with this proposal, showing that transcripts vary in their sensitivity to DDX3X/Ded1 depending on the extent of predicted structure in their 5’ UTRs (18, 20, 21, 22). DDX3X/Ded1 also associates with the eIF4F translation complex, which binds to the mRNA early in PIC assembly, and it appears to have a role in this process, again in an mRNA-specific fashion (19, 23, 24, 25, 26).

Interestingly, DDX3X/Ded1 can also act as a translation repressor (23, 27). In some cases, this may be a direct effect on translation, particularly in cellular stress, as we recently showed in conditions in which the target-of-rapamycin pathway is repressed (28). In addition, however, DDX3X/Ded1 is associated with stress granules (SGs), cytoplasmic accumulations of RNAs, and RNA-binding proteins that are believed to serve as sorting and storage sites for mRNAs during conditions of translation repression (29, 30, 31). DDX3X/Ded1 appears to play important roles in the formation and disassembly of SGs, though the cellular consequences of this function are not completely clear (23, 32). These effects appear to be primarily mediated through the N- and C-terminal regions of DDX3X/Ded1, which comprise low-complexity domains that contribute to liquid–liquid phase separations (33, 34). The medulloblastoma-associated mutations in *DDX3X* are not found in these regions, however.

Several studies have examined the effects of the *DDX3X* mutations identified in medulloblastoma. Despite the high frequency of mutations in the Wnt subtype of medulloblastoma and a reported direct effect on Wnt signaling by DDX3X (35), only minimal effects on Wnt signaling were observed with the mutants in reporter assays (10). Noting that many of the mutations are located in conserved regions of the helicase core (Fig. 1, *A* and *B*), two additional studies identified biochemical defects in a subset of mutants, focusing in particular on a few mutants with severe phenotypes *in vitro* and *in vivo* (36, 37). Further implicating translational defects in DDX3X-driven oncogenesis, Valentin-Vega and colleagues showed both an increase in SG formation and a general decrease in translation when a subset of *DDX3X* mutants were overexpressed in mammalian cell lines (38). Finally, hinting that the translational effects of the *DDX3X* mutations may be complex, ribosome profiling of cells expressing a medulloblastoma-associated *DDX3X* mutant showed some mRNA-specific effects, particularly on stress-related genes (39).

This previous work has contributed greatly to our understanding of the role of DDX3X/Ded1 in medulloblastoma and cancer generally. A limitation of these studies, however, is that they have generally focused on a small subset of the 42 distinct mutation sites that have been identified, largely for technical reasons. Therefore, it is unclear to what extent the results are representative or broadly applicable to the identified *DDX3X* mutations as a whole. Here, we have begun to address this question by taking advantage of the genetic tractability of budding yeast to construct a full set of mutations in *DDX3X/DED1* at all 42 identified sites (Fig. 1*A* and Table 1). The *S. cerevisiae* model system does not lend itself to detailed pathological analysis; however, since DDX3X and Ded1 are highly conserved in both sequence and function (13, 40), we conducted a comparison of molecular and cellular defects in the mutants. After an initial screening of the full set of mutants, we further characterized the translational and biochemical defects in a large, representative subset that reflects the spectrum of effects produced by the mutations. This analysis revealed that while some mutants had significant defects in bulk translation and particular biochemical properties, the most common and well-correlated defects in medulloblastoma-associated mutants of *DDX3X/DED1* were in specific translation processes, suggesting that these should be a focus in future studies of DDX3X-driven oncogenesis.

## Results

### *DDX3X* medulloblastoma-associated mutations inhibit growth when introduced into *DED1*

To characterize the impact of the medulloblastoma-associated mutations on cellular function, we began our study by mutating yeast *DED1* at the 42 conserved sites identified in medulloblastoma patient sequencing studies (Fig. 1, *A* and *B* and Table 1) (8, 9, 10, 11). Because *DED1* is an essential gene in yeast, these *ded1-mam* (*medulloblastoma-associated mutation*) alleles were introduced into *ded1-null* yeast on single-copy *CEN* plasmids *via* plasmid shuffle, replacing wild-type *DED1* as the only copy in cells. Following successful replacement, 9 of the 42 *ded1-mam* mutations resulted in lethality (Table 1). The lethal mutations were located across the helicase core and in different conserved motifs. This result is consistent with the lethality of several other known *ded1* point mutations in the conserved DEAD-box motifs (16, 23, 24). Furthermore, two of the lethal mutants (G261V and G284E) were previously shown to be incapable of complementing a temperature-sensitive *ded1* mutant in *Saccharomyces pombe* and had severe defects in enzymatic activity (36). The lethality of these *ded1-mam* mutants suggests an acute loss of DDX3X/Ded1 function for a subset of the medulloblastoma-associated mutations (see Discussion).

The viable *ded1-mam* mutants were further studied to determine if other growth defects were present. These mutants were screened for changes in growth on nutrient-rich agar at 16, 25, 30, and 37 °C (Fig. 1*C* and Table 1). Many of the medulloblastoma-associated mutants displayed temperature-dependent growth inhibition, while some mutants had no noticeable defect. Of the 33 viable *ded1-mam* mutants, 19 showed temperature-sensitive growth defects, including 16 cold-sensitive and 3 heat-sensitive (Table 1). Overall, growth inhibition or lethality occurred in two-thirds (28 out of 42) of the medulloblastoma-associated *DDX3X/ded1* mutants.

To further examine the effects of the *ded1-mam* mutations on DDX3X/Ded1 function, we took advantage of the ability of wild-type *DED1* to inhibit growth when highly overexpressed with a galactose-inducible promoter (23, 24, 41). Previously, the ability of *ded1* mutations to suppress this effect by rescuing growth correlated with effects on Ded1 function in translation and SG dynamics (23). Therefore, we transformed wild-type cells with galactose-inducible *ded1-mam* mutants and assayed their growth on galactose. Compared with wild-type *DED1*, all of the *ded1-mam* mutants tested had reduced ability to inhibit growth when overexpressed (Fig. S1*A*), even those without significant growth defects when expressed as the only copy at normal levels (*A184P*, *A352S*). This result suggests that all of the medulloblastoma-associated mutations affect DDX3X/Ded1 function in yeast.

Overall, these growth phenotypes suggest that the medulloblastoma-associated point mutations are sufficient to alter DDX3X/Ded1 function in yeast, validating the use of *S. cerevisiae* as a model for understanding the functional consequences of these mutations. Furthermore, the temperature-sensitive mutant alleles in particular allow for further study of the cellular and biochemical basis for the observed defects by shifting cells between permissive and nonpermissive conditions.

### The *ded1-mam* phenotypes are mostly recessive and are the result of altered Ded1 function

**Figure 2:**
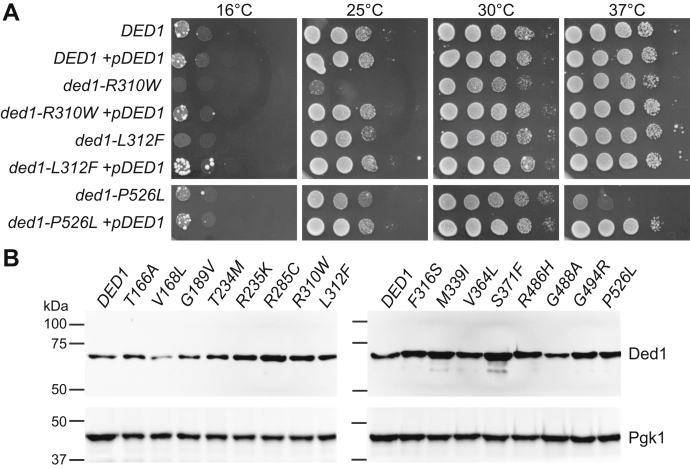
**Effects of expression level for medulloblastoma-associated mutations in *DDX3X/DED1*.***A*, wild-type *DED1* cells and the indicated *ded1-mam* mutants, containing either wild-type *DED1* on a low-copy plasmid or an empty control plasmid, were grown on selective media at 16, 25, 30, and 37 °C. Representative 5× serial dilutions of cells are shown. *B*, protein extracts from mid-log wild-type *DED1* cells and the indicated *ded1-mam* mutants were run on SDS-PAGE and blotted using anti-Ded1 and anti-Pgk1 (loading control) antibodies. Representative images are shown.

In order to better understand how the medulloblastoma-associated mutations in *DDX3X/DED1* contribute to *in vivo* phenotypes, we tested whether the growth defects of the *ded1-mam* mutants were dominant or recessive *via* two different methods. First, we coexpressed wild-type *DED1* on a low-copy plasmid in the *ded1-mam* mutants, which rescued growth to wild-type levels in all strains tested (Fig. 2*A* and data not shown). Second, we transformed plasmids expressing wild-type *DED1* or *ded1-mam* mutants into wild-type cells. Cells containing mutant plasmids generally grew similarly to cells containing either *DED1* plasmids or control plasmids, except for cells transformed with *ded1-T234M*, which had growth defects relative to control cells (Fig. S1*B*). Overall, these results suggest that the *ded1-mam* mutants function in a recessive manner, although a few (such as *T234M*) may display some dominant effects.

Next we examined protein levels of the *DDX3X/ded1* mutants. Although no gene amplifications or deletions of *DDX3X* have been reported in medulloblastoma, it remains possible that the identified mutations may affect protein stability. For instance, Valentin-Vega *et al.* (38) reported moderate increases in the protein levels of several *DDX3X* mutants in medulloblastoma patient samples. We therefore conducted western blotting of protein extracts from *ded1-mam* mutant cells. In general, the mutant cells showed Ded1 levels similar to wild-type (Fig. 2*B* and data not shown). Moderate increases or decreases were observed in a few mutants, including *ded1-S371F* and *ded1-V168L*, respectively, perhaps indicating that changes in protein level may contribute to the phenotypes of some mutants; however, these differences were not consistent with the severity of growth defects (Fig. 2*B* and Table 1). These results indicate that the phenotypic defects we observe in *ded1-mam* cells are likely due to defects in DDX3X/Ded1 function rather than changes in expression.

In order to further address whether protein levels play a role in the *ded1-mam* mutants, we utilized a high-copy plasmid vector (*2μ*) that results in increased expression of *DED1*, but at lower levels than the galactose-inducible constructs. Overexpressing wild-type *DED1 via* the *2μ* vector in cells lacking endogenous *DED1* had only a small effect on growth (Fig. S1*C*), suggesting that the growth defects observed in *ded1-mam* mutants are unlikely to be the sole result of moderate increases in protein level. Interestingly, however, *2μ*-mediated overexpression of several of the *ded1-mam* mutants showed better growth than low-copy (CEN) expression of the mutant (Fig. S1*C*), perhaps indicating that the residual activity of the mutants can partially restore function through mass-action.

### Medulloblastoma-associated mutations in *DDX3X/DED1* cause variable defects in general translation

We next sought to identify which cellular and molecular processes are affected by the medulloblastoma-associated mutations in *DDX3X/DED1*. While previous studies have identified defects in a select few mutants, it is unclear whether they are generally common to the spectrum of *DDX3X/DED1* mutations, and thus the identified phenotypes may not be key contributors to medulloblastoma pathology. Here, in our more broad-based approach, we reasoned that molecular and cellular defects that are: (1) the most broadly common across the range of different mutations, and (2) the best correlated with the observed growth defects in the *ded1-mam* mutants, are the most likely to be critical functional changes both in yeast and, because of the functional conservation of DDX3X/Ded1, in medulloblastoma. To conduct this analysis, we examined changes in known DDX3X/Ded1-dependent processes in representative subsets of 10 to 14 of the viable mutants from the *ded1-mam* collection, including multiple mutants with varying levels of growth defects, located in different regions of the coding sequence.

**Figure 3:**
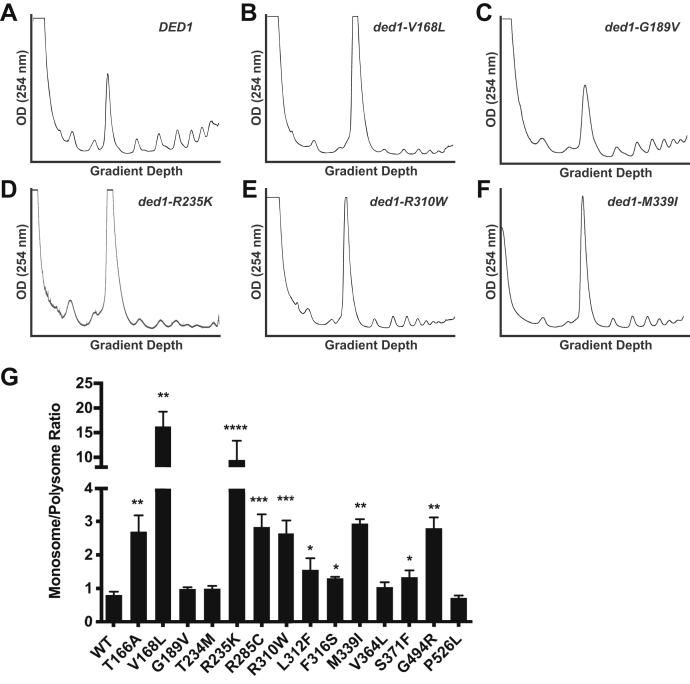
**Medulloblastoma-associated mutations in *DDX3X/DED1* cause variable defects in general translation.***A*–*F*, polyribosomal profiles of the indicated wild-type and *ded1-mam* mutants were generated by subjecting cell lysates to 7 to 47% sucrose density centrifugation and optical density analysis at 254 nm. Cells were incubated for 60 min before harvest at a nonpermissive temperature (18 °C for most mutants, *ded1-P526L* at 37 °C). Profile shown for wild-type *DED1* control (*A*) was incubated at 18 °C, although the profile at 37 °C was not significantly different. *G*, the monosome/polysome (M/P) ratios were determined by comparing the sum of the areas of the polysome peaks with the area of the monosome peak. Each M/P ratio shown is the mean and SEM from three to five independent trials. ∗*p* < 0.05, ∗∗*p* < 0.01, ∗∗∗*p* < 0.001, ∗∗∗∗*p* < 0.0001 *versus* wild-type *DED1*.

Because DDX3X/Ded1 is well characterized as a translation factor, we first examined whether the medulloblastoma-associated mutations caused defects in translation generally. To assess the bulk translation status of *ded1-mam* cells, we utilized sucrose density centrifugation to generate polyribosomal profiles of wild-type and mutant cells grown in nonpermissive conditions (examples in Fig. 3, *A*–*F*). From the profiles, we then calculated the 80S monosome-to-polyribosome (M/P) ratio for each sample, where a higher ratio would indicate a greater reduction in translation compared with wild-type cells (Fig. 3*G*). Most of the *ded1-mam* mutants (10 of 14 tested) had a significantly increased M/P ratio compared with wild-type cells (which had an M/P ratio of 0.8), indicating a defect in general translation in these cells. However, the level of defect varied substantially from an M/P ratio of 20.8 to close to wild-type, while four mutants did not show a significant defect, and three others only displayed a minor defect. Importantly, the M/P ratio in the *ded1-mam* mutants did not correlate well with growth phenotypes, as substantially different M/P defects were observed in mutants with similar growth (*e.g.*, *ded1-R235K versus* -*S371K* or -*T234M versus* -*G494R*, see also Fig. 6). Therefore, these results suggest that changes in general translation are not the primary effect of the medulloblastoma-associated mutations in yeast.

In order to further examine the links between DDX3X/Ded1 function, translation, and cell growth, we took advantage of a strain with a Tet-repressible *DED1* allele that allows for titration of Ded1 cellular protein levels (42). When *TET*_*off*_*-DED1* cells were treated with increasing levels of doxycycline, Ded1 protein levels decreased correspondingly (Fig. S2*A*). Likewise, the ability of *TET*_*off*_*-DED1* cells to grow either on plates or in liquid culture in the presence of doxycycline progressively decreased in a similar manner (Fig. S2, *B* and *C*). When polyribosomal profiling was performed on these cells, a similar effect was observed, with a progressive decrease in translation as Ded1 protein levels dropped (Fig. S2*D*). These results indicate that cellular growth and translation are normally highly correlated with DDX3X/Ded1 activity, as even a moderate reduction in Ded1 levels induced a similar reduction in translation and growth. Importantly, the medulloblastoma-associated mutations in *DDX3X/DED1* did not show a similar tight correlation, suggesting that these are not “simple” hypomorphic mutations that globally reduce DDX3X/Ded1 activity in translation in a straightforward manner. Rather, the mutations are likely to have more specific effects on translation that affect the expression of critical genes, causing the observed growth phenotypes.

### Medulloblastoma-associated mutations in *DDX3X/DED1* cause defects in translation of structured mRNAs and induce stress granules

**Figure 4:**
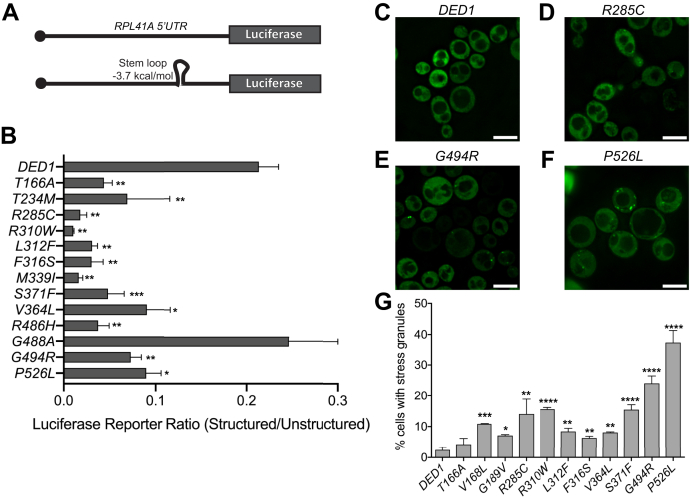
**Medulloblastoma-associated mutations in *DDX3X/DED1* have shared defects in 5’UTR scanning and stress granule formation.***A*, schematic of the unstructured (*top*) and structured (*bottom*) 5’UTR luciferase reporters. The 5’UTRs are derived from that of *RPL41A*, lengthened to 91 nt, with a stem loop inserted in the structured reporter. *B*, wild-type *DED1* and *ded1-mam* mutant cells containing these reporters were shifted to a nonpermissive temperature as in Figure 3 for 4 h, and cell extracts were subjected to luciferase assays. The ratio of luciferase activity between the structured and unstructured reporters is shown. *C*–*F*, wild-type *DED1* and the indicated *ded1-mam* mutant cells expressing *PAB1-GFP* as a marker for stress granules were shifted to a nonpermissive temperature as in Figure 3 for 2 h, and cells were imaged. The small GFP-positive foci represent stress granules. *G*, wild-type *DED1* and *ded1-mam* mutant cells were treated as above, and percentage of cells containing stress granules in each strain was determined. For (*C*) and (*G*), the mean and SEM from 3 to 11 independent trials is shown with ∗*p* < 0.05, ∗∗*p* < 0.01, ∗∗∗*p* < 0.001, ∗∗∗∗*p* < 0.0001 *versus* wild-type *DED1*. GFP, green fluorescent protein.

While the effects on bulk translation in the *ded1-mam* mutants fail to meet both of the above criteria for critical phenotypes, defects that alter translation of subsets of mRNAs are still potential candidates. Notably, DDX3X and Ded1 both have well-characterized roles in facilitating start site scanning in 5’UTRs with significant secondary structure, leading to hyperdependence on DDX3/Ded1 for translation of mRNAs containing such 5’UTRs (18, 19, 21, 22). We thus hypothesized that the medulloblastoma-associated mutations in *DDX3X/DED1* may be specifically affecting this function. To examine this process, we obtained previously published luciferase reporters with 5’UTRs derived from *RPL41A* that either contain a stem loop (ΔG_free_ = −3.7 kcal/mol) or are unstructured (Fig. 4*A*) (21). In the prior study, the stem-loop-containing reporter was translated less well than the unstructured one in wild-type cells, and this difference was exacerbated by a temperature-sensitive *ded1* mutant. Here, we transformed these reporters into the *ded1-mam* mutants, incubated the cells at a nonpermissive temperature, and performed luciferase assays (Fig. 4*B*). Consistent with published reports, the ratio of luciferase activity in the structured *versus* unstructured reporters was 0.21 in wild-type control cells. Strikingly, 12 of the 13 *ded1-mam* mutants tested had a significant defect in translation of the structured reporter, and most showed structured *versus* unstructured ratios of less than 0.05 (Fig. 4*B*). The defects also correlated fairly well with the growth phenotypes of the mutants (see Fig. 6). In fact, the one mutant without a significant effect on the structured reporter, *ded1-G488A*, also did not have a detectable growth phenotype (Table 1). These results indicate that the medulloblastoma-associated mutations in *DDX3X/DED1* have a shared, specific effect on translation of mRNAs containing structured 5’UTRs, suggesting that this defect may be crucial for medulloblastoma progression.

DDX3X/Ded1 has also been linked to the dynamics of stress granules (SGs), and Valentin-Vega *et al.* (38) reported that overexpression of several medulloblastoma-associated *DDX3X* mutants induced SGs in mammalian cells. To test the possibility that *ded1-mam* mutants similarly induce SGs, we examined whether a subset of *ded1-mam* cells containing green fluorescent protein-tagged *PAB1* as a SG marker formed constitutive SGs. As expected, only a small percentage of wild-type cells (2.5%) contained green fluorescent protein-positive foci, indicating the presence of SGs (Fig. 4, *C* and *G*). In contrast, all 11 of the *ded1-mam* mutants tested showed at least a slight increase in the percentage of cells containing foci, with all except *ded1-T166A* significantly increased compared with wild-type (Fig. 4, *D*–*G*). Two mutants, *ded1-P526L* and -*G494R*, showed a more substantial increase with 37.2% and 24.1%, respectively (Fig. 4, *E*–*G*). Interestingly, these two mutants also had relatively weak defects in scanning structured 5’UTRs (Fig. 4*B*), perhaps indicating that constitutive SG formation can exacerbate the effect of the scanning defects in causing translational changes and growth phenotypes. Overall, the increases in SG formation do not correlate well with the *ded1-mam* growth phenotypes, which suggests that these effects are less critical than the scanning defects (see Fig. 6). However, given that SGs were increased in most of the mutants tested suggests that this shared phenotype may still have some relevance in medulloblastoma progression.

### The DDX3X/Ded1 mutants have moderate but variable defects in enzymatic activity

**Figure 5:**
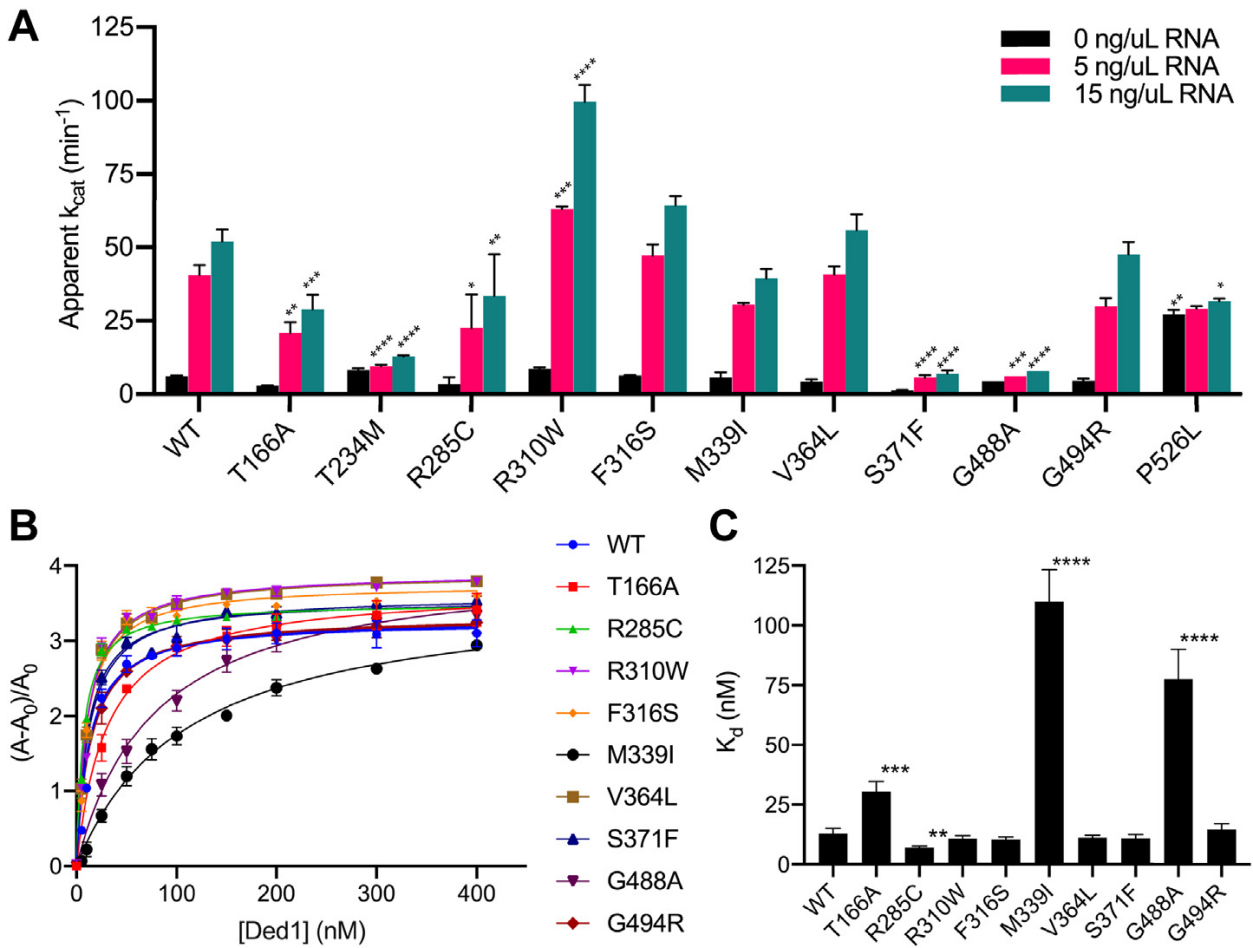
**Medulloblastoma-associated Ded1 mutants do not have consistent defects in ATPase activity or RNA binding.***A*, ATPase activity of purified recombinant His-Ded1 protein (WT) and the indicated ded1 mutants was determined with a colorimetric PK/LDH-coupled assay in the presence of 0, 5, and 15 ng/μl total cellular RNA. The apparent k_cat_ (min^−1^) is shown as the mean and SEM of 3 to 6 independent trials. *B* and *C*, equilibrium RNA binding activity for purified recombinant Ded1 protein (wild-type and the indicated mutants) was determined by fluorescence anisotropy with a fluorescein-labeled RNA oligonucleotide in the presence of ADP-BeF_x_. Curve fitting (*B*) and apparent binding affinities (*C*) were calculated *via* a single-binding site model from 3 to 6 independent trials. ∗*p* < 0.05, ∗∗*p* < 0.01, ∗∗∗*p* < 0.001, ∗∗∗∗*p* < 0.0001 *versus* wild-type Ded1 protein.

In order to better understand the biochemical basis of the effects of the medulloblastoma-associated mutations in *DDX3X/DED1*, we purified His-tagged, recombinant wild-type and mutant Ded1 protein and subjected them to two different *in vitro* assays. First, we conducted coupled colorimetric ATPase assays as a gauge of the mutant proteins’ enzymatic activity (24, 43). Consistent with previous reports (24, 44), wild-type Ded1 displayed highly RNA-dependent ATPase activity, with low activity without RNA and nearly ninefold higher activity (apparent k_cat_ of 52 min^−1^) in the presence of 15 ng/μl total RNA (Fig. 5*A*). The ded1 mutants tested yielded a range of results, from low activity for ded1-S371F (k_cat_ = 7 min^−1^ with 15 ng/μl RNA) to activity greater than wild-type for ded1-R310W (k_cat_ = 100 min^−1^ with 15 ng/μl RNA). Most commonly, the ded1-mam mutants showed a mild-to-moderate defect in ATPase activity (5 out of 11 tested), and none of the mutations completely abrogated activity. However, the severity of defect did not correlate well with growth phenotypes. For example, the ded1-G488A mutant, which had significantly reduced ATPase activity, did not cause detectable growth defects in cells, while ded1-R310W had robust ATPase activity *in vitro* yet had substantial growth defects (Table 1 and Fig. 5*A*).

As a second test for biochemical defects, we assayed RNA binding by the mutant proteins using fluorescence anisotropy with a labeled oligonucleotide (Fig. 5, *B* and *C*) (24). In the presence of ADP-BeF_x_, a nonhydrolyzable ATP analog, wild-type Ded1 bound quite tightly to RNA (apparent K_d_ = 13 nM), consistent with previous reports (24). Most of the mutants tested showed similar RNA affinities, although ded1-M339I (K_d_ = 110 nM) and -G488A (K_d_ = 78 nM) were notable exceptions (Fig. 5*C*). For ded1-G488A, this result is consistent with the defect in ATPase activity in Figure 5*A*. Somewhat surprisingly, however, ded1-M339I displayed only a small, nonsignificant change in RNA-dependent ATPase activity. The RNA concentrations in the two biochemical assays are not directly comparable (total cellular RNA *versus* an *in vitro* synthesized oligonucleotide), but the relative ATPase activities at 5 and 15 ng/μl suggest that the ATPase assays were performed near RNA saturation conditions for wild-type Ded1 and most of the mutants, including ded1-M339I, which may explain the difference between the assay results. It is also possible that ded1-M339I and ded1-G488A are not fully functional when purified *in vitro* or that other differences between the assays (*e.g.*, binding ATP *versus* ADP-BeF_x_) are responsible for the differences between the two assays. Likewise, ded1-S371F, which had reduced ATPase activity, showed an RNA affinity similar to wild-type (K_d_ = 11 nM), indicating that a different biochemical activity is defective in this mutant. Overall, these results argue that significant defects in RNA binding are generally not a major contributor to mutant pathology. Taken together with the ATPase results, they suggest that the basis of the mutants’ phenotypes and contribution to pathology is not limited to defects in a single biochemical activity, but rather that alterations in multiple activities can cause the observed phenotypes.

### The phenotypes of the medulloblastoma-associated mutants of *DDX3X/DED1* correlate best with defects in translating mRNAs with structured 5’UTRs

In this study, we have characterized the functional and biochemical effects of medulloblastoma-associated mutations in *DDX3X/DED1*. The results for the most well-characterized mutants are summarized in Table S1. One advantage of the broad-based approach taken here is that it allows for analysis of the correlation between different results, particularly between the observed growth phenotypes and the functional defects. Furthermore, such an analysis is one of the criteria, as stated above, for evaluating the contribution of different phenotypes. We utilized two different methods to identify correlations.

**Figure 6:**
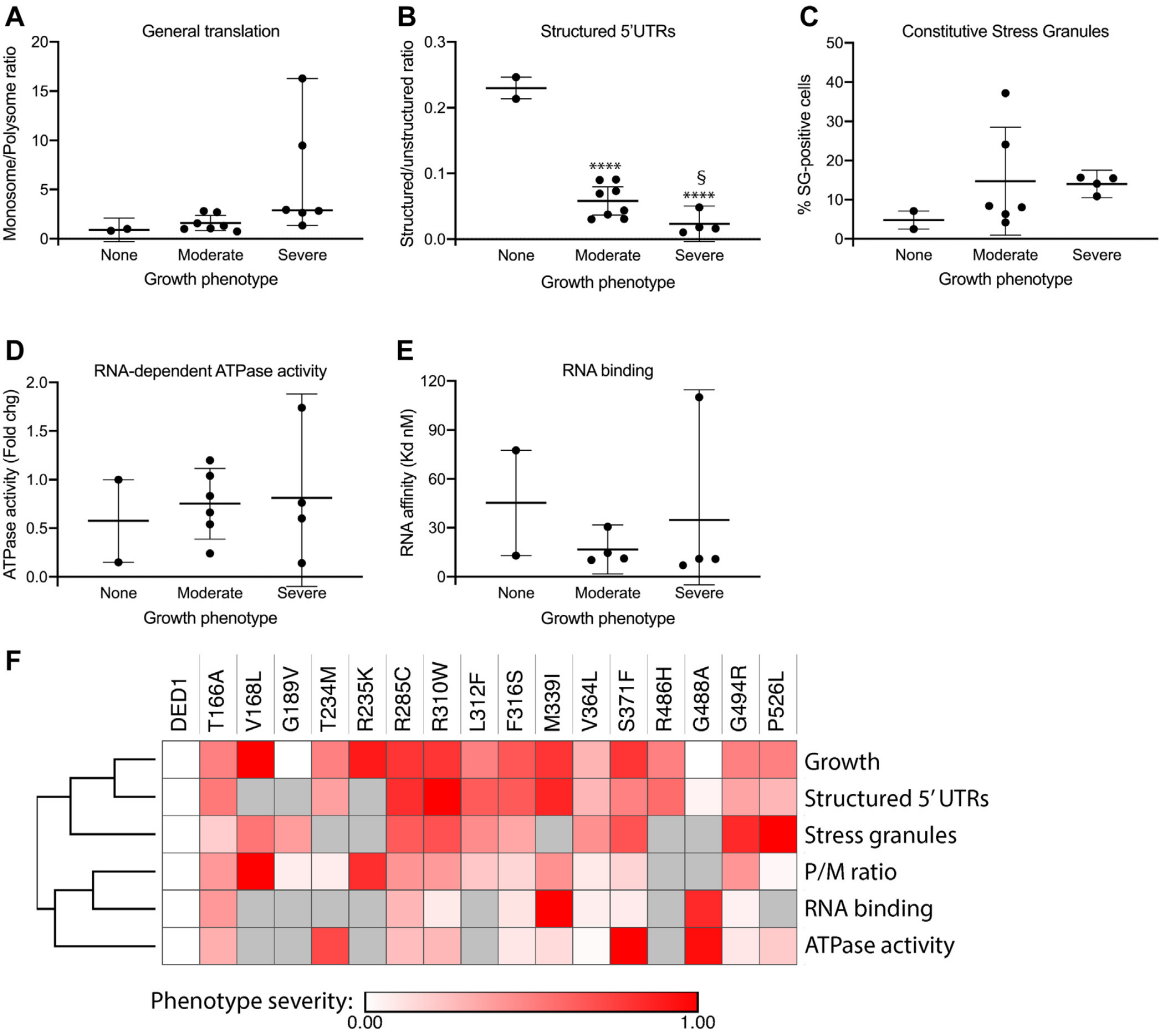
**Specific translation defects in scanning 5’UTRs in *ded1-mam* mutations correlate best with growth phenotypes.***A*–*E*, the phenotype of each extensively-tested *ded1-mam* mutant in each of the five phenotypes shown (general translation, structured 5’UTRs, constitutive stress granules, ATPase activity, and RNA binding) was quantified. The mutants were then sorted by growth phenotype into no growth defects (None), mild-to-moderate growth defects (Moderate), and substantial growth defects (Severe) and graphed compared with the indicated phenotype. Whisker plots indicate the median and 95% confidence interval for each data set. ∗∗∗∗*p* < 0.0001 *versus* None, ^§^*p* <0.05 *versus* Moderate. *F*, a heatmap was generated by scaling the magnitude of phenotypes for each *ded1-mam* mutant to a similar range, and clustering was determined by average Euclidean distance. Phenotype severity is shown by brighter *red color*, and values that were not determined are in *gray*.

First, the *ded1-mam* mutants were binned by their overall growth phenotype (none, moderate, or severe), and their phenotypes were compared in each of the five major functions tested (total translation, structured 5’UTR scanning, SG formation, ATPase activity, and RNA affinity) (Fig. 6, *A*–*E*). For total translation (as measured by M/P ratio in polyribosome profiles), there was a weak trend toward more severe translation defects as mutant growth decreased (Fig. 6*A*). However, this effect was largely driven by two mutants with large defects (*ded1-V168L* and -*R235K*), while most of other mutants had only minor defects. By contrast, for scanning, there was a much stronger trend toward more severe defects as growth decreased, with less variability (Fig. 6*B*). SG formation, ATPase activity, and RNA binding affinity did not show any clear trends, other than a minor increase in constitutive SGs in mutants in the “severe” growth defect category compared with “none” (Fig. 6, *C*–*E*).

For the second analysis, we conducted hierarchical clustering of the six major phenotypes (the five functions above plus growth) across the set of mutants (Fig. 6*F*). The clustering revealed that the growth phenotypes correlated most strongly with the scanning defects, while the SG phenotypes were the next most similar. Interestingly, in this analysis, the polyribosome profile defects were more distant from growth, weakly correlating with the biochemical phenotypes. These results strongly suggest that a specific translation defect, scanning through structured 5’UTRs, is primarily responsible for the growth defects in the *ded1-mam* mutants, and thus we suggest it is likely to be the most critical driver of cellular changes due to *DDX3X/DED1* mutations in medulloblastoma.

## Discussion

Determining the role of *DDX3X* mutations in medulloblastoma is a challenging problem. The lack of identified frameshift or nonsense mutations strongly suggests that straightforward inactivation of the gene does not promote progression of medulloblastoma. Compounding this, the number of different mutation sites makes identifying the critical phenotypes more difficult, as it is possible that an individual mutant may induce phenotypes that are not relevant to the pathology. These complexities suggest the benefit of a broad-based approach in which larger sets of mutants are studied in detail; however, there are practical limitations to such an approach in most mammalian systems. Therefore, in this study, we took advantage of the yeast model system, allowing characterization of all 42 identified mutation sites, which had not been done previously. Despite the inability to study cancer progression *per se* in yeast, the high degree of sequence and functional conservation of *DED1* and *DDX3X* (Fig. 1*B* and (40)) argue strongly that functional defects in the mutants are likely to be conserved. This approach is further validated by the observation that 41 of the sites are identical in budding yeast and humans in wild-type alleles and by our data showing that two-thirds of the *ded1-mam* mutants have an observable growth phenotype in yeast (Fig. 1*C* and Table 1). This broad-based strategy allowed us to identify which phenotypes are shared by the different mutations and to correlate the observed functional and biochemical defects with the growth phenotypes. Notably, we found that a specific defect in translation of mRNAs with structured 5’UTRs (Figs. 4, *A* and *B* and 6) was the most common and well-correlated phenotype of those examined. These results indicate that this defect is highly likely to be the major functional consequence of the mutations in yeast, and therefore, we suggest it may also be a key contributor to cancer progression in medulloblastoma. In contrast, functions in bulk translation and SG formation, as well as the specific biochemical activities of ATP hydrolysis and RNA binding, were not widely shared by all mutants nor correlated with growth (Figs. 3, 4, *C*–*G*, 5 and 6), suggesting that these defects may be less critical determinants in medulloblastoma.

Although some *ded1-mam* mutants did show general translation defects, this was not consistently the case, and furthermore, the observed defects correlated poorly with the effects on growth (Figs. 3 and 6). Therefore, we suggest that bulk translation reduction is less likely to be a major contributor to DDX3X-mediated oncogenesis. We note that Valentin-Vega *et al.* (38) proposed that global translation impairment is an important feature of *DDX3X* mutations in medulloblastoma, based on analysis of a more limited set of mutants in mammalian cell culture. Two of the three translationally characterized mutations in that study (G302V and G325E) were lethal in our hands, consistent with the severe phenotypes described for these mutants (36, 38). Highlighting the importance of a broad approach, however, they were not representative of the mutant phenotypes as a whole. Rather, Figure 3, with translation data from 14 different mutants, clearly shows the variability in translation defects in the different mutants. Instead of a general translation defect, our data suggest that the major functional consequence of mutations in *DDX3X* is to promote cancer through more specific effects on translation.

Several studies have demonstrated that mRNAs vary in their dependence on Ded1 for translation (21, 22, 25). Here, defects in AUG start site scanning through structured 5’UTRs were the most consistent defect in the medulloblastoma-associated mutant cells, and this defect showed the best correlation with effects on growth (Figs. 4*B* and 6). These results are also consistent with the promotion of scanning as the most well-established function of DDX3X/Ded1 (18, 20, 21, 22). Moreover, scanning appears to be quite sensitive to even small perturbations in DDX3X/Ded1 activity (21, 22). The connection between scanning defects and cancer progression is less clear. Oncogenic mRNAs often contain longer, more structured 5’UTRs than the average (45), and reduced DDX3X activity would decrease translation of these mRNAs, other things equal. However, more complex effects as a result of decreased scanning have been demonstrated in other *ded1* mutants, including increased translation at alternative translation initiation sites and upstream open reading frames in a subset of mRNAs (22). Thus, we speculate that similar mechanisms may regulate DDX3X translation targets in medulloblastoma.

In addition to scanning defects, we observed an increase in constitutive stress granules in most of the *ded1-mam* mutants tested, though this phenotype was minimal for some mutants and did not correlate well with effects on growth (Figs. 4*G* and 6*C*). It remains possible, however, that changes in SG dynamics play a role in DDX3X-mediated medulloblastoma. Notably, increased SGs were observed previously upon overexpression of several *DDX3X* mutants in HeLa cells (38), including two (R351W and L353F) that also had increased SGs in yeast (Fig. 4*G*). The connections between SGs and cancer are not well understood and may be specific to the cancer type, the SG components present, and the method of stress induction (30, 46). Interestingly, stress response pathways, which are often deregulated in cancer cells, result in changes in translation (47, 48, 49). Indeed, we recently showed that Ded1 has a role in the translational response to cell stress (28), and Oh *et al.* (39) observed stress-dependent changes in candidate mRNA targets in HEK293 cells overexpressing a *DDX3X* medulloblastoma-associated mutant. Follow-up studies that expand this work may be able to further elucidate the connections between DDX3X/Ded1, stress, and cancer.

Despite the widely shared and well-correlated scanning defect in the *ded1-mam* mutants, the medulloblastoma-associated mutations in *DDX3X/DED1* had variable defects in growth, and many also showed additional functional defects. This variability of the effect of the mutations is in agreement with previous studies, and the mutations with the most striking phenotypes in these *in vitro* and overexpression studies (*e.g.*, G302V, G325E, and R534H) were lethal *in vivo* in our hands in yeast (36, 37, 38). Whether these differences between individual mutants have consequences in medulloblastoma is unclear. There are no obvious relationships between the phenotypes we and others have observed and the locations of the mutations in conserved DEAD-box protein motifs (12), nor is there a noticeable correlation with the presumed zygosity of the *DDX3X* mutations in tumors (inferred from the fraction of mutant *versus* wild-type sequences obtained from available patient data (8, 9, 11)). Interestingly, studies of *DDX3X* mutations identified in natural killer/T-cell lymphoma and intellectual disability syndromes have suggested that the mutations in these diseases are quite severe and include frameshifts and nonsense mutations that are not present in medulloblastoma (50, 51, 52, 53). In contrast, the medulloblastoma-associated mutations appear to have a unique molecular pathology wherein the specific scanning defect is most critical to disease progression, but a range of other defects are also tolerated. More studies of DDX3X molecular pathology will be needed to resolve these issues.

This study of the molecular and cellular defects of *ded1-mam* mutants in yeast identified which of the known functions of DDX3X/Ded1 are most likely responsible for the observed growth phenotypes. Therefore, we suggest that the mutations may have similar functional effects in medulloblastoma, providing potential insight into its molecular pathology. Using *S. cerevisiae* as a model for human cancer does have limitations, however, indicating the need for further studies. These limitations include the lack of the Wnt signaling pathway, which is canonically affected in the Wnt-subtype of medulloblastoma. DDX3X has been shown to regulate this pathway through activation of casein kinase 1ε (35), although several DDX3X medulloblastoma-associated mutants did not have a major effect on Wnt transcriptional targets when tested previously (10). Furthermore, a fair number of the *ded1-mam* mutants did not show a significant growth defect in yeast, such as the apparently well-tolerated G488A, which may indicate that human cells are even more sensitive to *DDX3X/DED1* perturbations than yeast. Therefore, it will be important for future studies to verify and expand our findings in mammalian cells and animal models. In turn, we suggest that our results provide potential direction to such studies, including a focus on scanning defects using the mutations that are the most representative of the full spectrum. In particular, since the *ded1-mam* mutations result in specific translation defects, a ribosomal profiling strategy using CRISPR/Cas9-generated cells and/or mice would be instrumental in identifying the affected mRNA targets. This knowledge could then be used to help understand what physiological processes are affected by the *DDX3X* mutations in the development of medulloblastoma and, ultimately, how its effects can be reversed by future therapeutics for the disease.

## Experimental procedures

### Strain and plasmid construction

Yeast strains and plasmids are listed in Tables S2 and S3. For all constructs, *ded1-mam* mutations were generated by site-directed mutagenesis of the appropriate wild-type *DED1* plasmid and confirmed by sequencing. The starting template plasmids were: pSW3619 for *CEN* plasmids, pRP2086 for Gal-inducible plasmids, pTB105 for *2μ* plasmids, and pSW3576 for recombinant expression plasmids. Yeast strains containing the *ded1-mam* mutants were generated by plasmid shuffle into the strain SWY4093 (*ded1::KAN* +*pDED1/URA3*). Yeast growth assays were performed by serial dilution as previously described (24).

### Western blotting

Blotting for Ded1 expression levels was carried out as described in (24). Crude cell lysates were prepared by lysis in NaOH and β-mercaptoethanol followed by trichloroacetic acid precipitation. Samples were separated by SDS-PAGE and blotted with specific antibodies toward Ded1 (VU 318, described in (54)) and Pgk1 (Life Technologies). HRP-conjugated secondary antibodies were used to visualize bands on a Sapphire biomolecular imager (Azure Biosystems).

### Polyribosome preparation

Polyribosome profiles were generated as described in (24). The indicated mutants were grown in rich media at 30 °C, then shifted to a nonpermissive temperature (18 °C or 37 °C in the case of *ded1-P526L*) for 1 h prior to harvest. Cells were lysed and subjected to 7 to 47% sucrose density centrifugation for 2.5 h at ∼178,000*g*, then samples were fractionated, and RNA curves were generated by monitoring absorbance at 254 nm. Monosome/polysome ratios were determined by comparing the area under the curve for the 80S peak with the sum of the polyribosome peaks in ImageJ (National Institutes of Health). Significance *versus* wild-type *DED1* was determined *via* Mann–Whitney nonparametric test in Prism (Graphpad).

### *TET*_*off*_*-DED1* analysis

The *TET*_*off*_*-DED1* was obtained from the Tet-Promoters Hughes Collection (Horizon). A range of doxycycline concentrations (0–0.5 μg/ml) was added to media in order to titrate Ded1 levels. For growth assays, doxycycline was continuously present throughout the assay, whereas for western blotting and polyribosome profiles, doxycycline was added 8 h before harvest. Growth curves were generated by diluting log-phase cells in selective media and incubating at 25 °C for 10.5 h. Optical density measurements were taken approximately every 90 min and fitted to an exponential growth equation in Prism. Significance was determined by Student’s paired *t*-test.

### Scanning assays

Scanning assays for structured 5’UTR sequences were carried out similarly to (21). Briefly, cells transformed with either the control unstructured 5’UTR reporter (pFJZ342) or the stem-loop-containing 5’UTR reporter (pFJZ623) were cultured in triplicate at 30 °C, then shifted to nonpermissive temperature (18 °C or 37 °C) for 4 h. Cell lysates were generated *via* bead beater in luciferase lysis buffer (25 mM Tris phosphate pH 7.8, 2 mM EGTA, 2 mM DTT, 0.5% Triton X-100, 10% glycerol). Luciferase assays were performed with standard luciferin reagent (Promega) on a Glomax 20/20 luminometer (Promega). Triplicates were averaged together for each biological replicate, and significance *versus* wild-type *DED1* was determined *via* Student’s unpaired *t*-test.

### Fluorescence microscopy

For SG analysis, the indicated strains were transformed with a plasmid expressing *PAB1-GFP* and *EDC3-mCherry* (pRP1657). Cells were cultured in selective media at 30 °C, then shifted to nonpermissive temperature (18 °C or 37 °C) for 2 h. Images were captured using a DeltaVision Elite inverted microscope (Applied Precision/GE Healthcare) with an Olympus 100× plan apo NA 1.4 objective and appropriate filter sets. Z-series data sets were collected with a pco.edge sCMOS camera at a step size of 0.4 μm. Postacquisition deconvolution was performed using SoftWorx software (Applied Precision). Z-series processing, quantitation, cropping, and sizing were completed in ImageJ/Fiji. Significance *versus* wild-type *DED1* was determined *via* Student’s unpaired *t*-test.

### ATPase activity assays

Recombinant His-Ded1 protein and mutant derivatives were generated as described in (24). Total RNA was purified from yeast *via* hot phenol extraction. ATPase assays were performed by PK/LDH-coupled colorimetric assay as in (24, 43). Reactions were conducted with 150 nM Ded1 or mutant protein, 0, 5, or 15 ng/μl total RNA, 1 mM ATP, 3 mM phosphoenolpyruvate, 210 μM NADH, and ∼17.5 units/ml PK/LDH mixture (Sigma) in buffer (20 mM HEPES pH 7.5, 40 mM NaCl, 3 mM MgCl_2_, 1 mM DTT, and 0.2 units/μl RNasin (Promega)). Reactions were started by the addition of ATP and the coupled colorimetric components to preincubated protein and RNA, then absorbance at 340 nm was read for 20 min on a Versamax microplate reader (Molecular Devices). Apparent k_cat_ was calculated from the absorbance signal decline to measure steady-state ATPase activity. Significance was determined by two-way ANOVA with Bonferroni correction.

### RNA-binding assays

RNA-binding experiments were carried out by fluorescence anisotropy with a 16-mer RNA oligomer (5’-AGCACCGUAAAGACGC-3’) labeled at the 5’ end with 6-carboxyfluorescein (IDT) as described in (24). Ded1 or mutant protein at the indicated concentrations (0–400 nM) was preincubated in the presence of 2 mM ADP-BeF_x_ (prepared as described in (55)) in buffer (20 mM HEPES pH 7.5, 40 mM NaCl, and 20% glycerol), and then 2 nM of the labeled RNA was added and incubated with the samples for 30 min. Polarized fluorescence was read on a Synergy HT plate reader (BioTek), and anisotropy values were calculated as (A − A_0_)/A_0_. Data were fitted to a one-site binding curve in Prism, and apparent K_d_ was calculated. Significant differences between wild-type and mutant K_d_ were determined by extra sum-of-squares F test.

### Correlation analysis

For the plots in Figure 6, *A*–*E*, each of the characterized strains (wild-type *DED1* and 16 *ded1-mam* mutants) was binned into one of three categories (none, moderate, or severe) based on their growth phenotypes as shown in Table 1. They were then graphed according to the quantitation of a given phenotype (*e.g.*, M/P ratio for general translation defects). The median and 95% confidence interval are shown. Significance was determined *via* one-way ANOVA with Fisher’s least significant difference test. For the hierarchical clustering analysis, each of the six phenotypes was first converted into a fold change and then to a log-2 change *versus* wild-type cells, including growth, where relative fitness was estimated given the results as determined in Table 1. Morpheus software (Broad Institute, https://software.broadinstitute.org/morpheus) was used to conduct hierarchical clustering among the six phenotypes by Euclidean distance. To normalize across phenotypes for display purposes, the log-2 changes were converted to a 0 to 1 scale; however, this normalization did not alter the clustering results.

## Data availability

All data are contained within the article or supplementary information. Original data will be shared upon request of the corresponding author.

## Conflict of interest

The authors declare that they have no conflicts of interest with the contents of this article.

## References

[1] Northcott P.A., Jones D.T., Kool M., Robinson G.W., Gilbertson R.J., Cho Y.J., Pomeroy S.L., Korshunov A., Lichter P., Taylor M.D., Pfister S.M. (2012). Medulloblastomics: The end of the beginning. Nat. Rev. Cancer.

[2] Blessing M.M., Alexandrescu S. (2020). Embryonal tumors of the central nervous system: An update. Surg. Pathol. Clin..

[3] Taylor M.D., Northcott P.A., Korshunov A., Remke M., Cho Y.J., Clifford S.C., Eberhart C.G., Parsons D.W., Rutkowski S., Gajjar A., Ellison D.W., Lichter P., Gilbertson R.J., Pomeroy S.L., Kool M. (2012). Molecular subgroups of medulloblastoma: The current consensus. Acta Neuropathol..

[4] Li Q., Dai Z., Cao Y., Wang L. (2018). Comparing children and adults with medulloblastoma: A SEER based analysis. Oncotarget.

[5] Ris M.D., Packer R., Goldwein J., Jones-Wallace D., Boyett J.M. (2001). Intellectual outcome after reduced-dose radiation therapy plus adjuvant chemotherapy for medulloblastoma: A Children's Cancer Group study. J. Clin. Oncol..

[6] Mabbott D.J., Penkman L., Witol A., Strother D., Bouffet E. (2008). Core neurocognitive functions in children treated for posterior fossa tumors. Neuropsychology.

[7] Hanzlik E., Woodrome S.E., Abdel-Baki M., Geller T.J., Elbabaa S.K. (2015). A systematic review of neuropsychological outcomes following posterior fossa tumor surgery in children. Childs Nerv. Syst..

[8] Jones D.T., Jager N., Kool M., Zichner T., Hutter B., Sultan M., Cho Y.J., Pugh T.J., Hovestadt V., Stutz A.M., Rausch T., Warnatz H.J., Ryzhova M., Bender S., Sturm D. (2012). Dissecting the genomic complexity underlying medulloblastoma. Nature.

[9] Kool M., Jones D.T., Jager N., Northcott P.A., Pugh T.J., Hovestadt V., Piro R.M., Esparza L.A., Markant S.L., Remke M., Milde T., Bourdeaut F., Ryzhova M., Sturm D., Pfaff E. (2014). Genome sequencing of SHH medulloblastoma predicts genotype-related response to smoothened inhibition. Cancer Cell.

[10] Pugh T.J., Weeraratne S.D., Archer T.C., Pomeranz Krummel D.A., Auclair D., Bochicchio J., Carneiro M.O., Carter S.L., Cibulskis K., Erlich R.L., Greulich H., Lawrence M.S., Lennon N.J., McKenna A., Meldrim J. (2012). Medulloblastoma exome sequencing uncovers subtype-specific somatic mutations. Nature.

[11] Robinson G., Parker M., Kranenburg T.A., Lu C., Chen X., Ding L., Phoenix T.N., Hedlund E., Wei L., Zhu X., Chalhoub N., Baker S.J., Huether R., Kriwacki R., Curley N. (2012). Novel mutations target distinct subgroups of medulloblastoma. Nature.

[12] Linder P., Jankowsky E. (2011). From unwinding to clamping - the DEAD box RNA helicase family. Nat. Rev. Mol. Cell Biol..

[13] Sharma D., Jankowsky E. (2014). The Ded1/DDX3 subfamily of DEAD-box RNA helicases. Crit. Rev. Biochem. Mol. Biol..

[14] Shen L., Pelletier J. (2020). General and target-specific DExD/H RNA helicases in eukaryotic translation initiation. Int. J. Mol. Sci..

[15] Aylett C.H., Ban N. (2017). Eukaryotic aspects of translation initiation brought into focus. Philos. Trans. R. Soc. Lond. B Biol. Sci..

[16] Chuang R.Y., Weaver P.L., Liu Z., Chang T.H. (1997). Requirement of the DEAD-box protein Ded1p for messenger RNA translation. Science.

[17] Lee C.S., Dias A.P., Jedrychowski M., Patel A.H., Hsu J.L., Reed R. (2008). Human DDX3 functions in translation and interacts with the translation initiation factor eIF3. Nucleic Acids Res..

[18] Lai M.C., Lee Y.H., Tarn W.Y. (2008). The DEAD-box RNA helicase DDX3 associates with export messenger ribonucleoproteins as well as Tip-associated protein and participates in translational control. Mol. Biol. Cell.

[19] Soto-Rifo R., Rubilar P.S., Limousin T., de Breyne S., Decimo D., Ohlmann T. (2012). DEAD-box protein DDX3 associates with eIF4F to promote translation of selected mRNAs. EMBO J..

[20] Berthelot K., Muldoon M., Rajkowitsch L., Hughes J., McCarthy J.E. (2004). Dynamics and processivity of 40S ribosome scanning on mRNA in yeast. Mol. Microbiol..

[21] Sen N.D., Zhou F., Ingolia N.T., Hinnebusch A.G. (2015). Genome-wide analysis of translational efficiency reveals distinct but overlapping functions of yeast DEAD-box RNA helicases Ded1 and eIF4A. Genome Res..

[22] Guenther U.P., Weinberg D.E., Zubradt M.M., Tedeschi F.A., Stawicki B.N., Zagore L.L., Brar G.A., Licatalosi D.D., Bartel D.P., Weissman J.S., Jankowsky E. (2018). The helicase Ded1p controls use of near-cognate translation initiation codons in 5' UTRs. Nature.

[23] Hilliker A., Gao Z., Jankowsky E., Parker R. (2011). The DEAD-box protein Ded1 modulates translation by the formation and resolution of an eIF4F-mRNA complex. Mol. Cell.

[24] Aryanpur P.P., Regan C.A., Collins J.M., Mittelmeier T.M., Renner D.M., Vergara A.M., Brown N.P., Bolger T.A. (2017). Gle1 regulates RNA binding of the DEAD-box helicase Ded1 in its complex role in translation initiation. Mol. Cell. Biol..

[25] Gupta N., Lorsch J.R., Hinnebusch A.G. (2018). Yeast Ded1 promotes 48S translation pre-initiation complex assembly in an mRNA-specific and eIF4F-dependent manner. Elife.

[26] Gulay S., Gupta N., Lorsch J.R., Hinnebusch A.G. (2020). Distinct interactions of eIF4A and eIF4E with RNA helicase Ded1 stimulate translation *in vivo*. Elife.

[27] Shih J.W., Tsai T.Y., Chao C.H., Wu Lee Y.H. (2008). Candidate tumor suppressor DDX3 RNA helicase specifically represses cap-dependent translation by acting as an eIF4E inhibitory protein. Oncogene.

[28] Aryanpur P.P., Renner D.M., Rodela E., Mittelmeier T.M., Byrd A., Bolger T.A. (2019). The DEAD-box RNA helicase Ded1 has a role in the translational response to TORC1 inhibition. Mol. Biol. Cell.

[29] Buchan J.R., Parker R. (2009). Eukaryotic stress granules: The ins and outs of translation. Mol. Cell.

[30] Anderson P., Kedersha N., Ivanov P. (2015). Stress granules, P-bodies and cancer. Biochim. Biophys. Acta.

[31] Protter D.S.W., Parker R. (2016). Principles and properties of stress granules. Trends Cell Biol..

[32] Shih J.W., Wang W.T., Tsai T.Y., Kuo C.Y., Li H.K., Wu Lee Y.H. (2012). Critical roles of RNA helicase DDX3 and its interactions with eIF4E/PABP1 in stress granule assembly and stress response. Biochem. J..

[33] Hondele M., Sachdev R., Heinrich S., Wang J., Vallotton P., Fontoura B.M.A., Weis K. (2019). DEAD-box ATPases are global regulators of phase-separated organelles. Nature.

[34] Iserman C., Desroches Altamirano C., Jegers C., Friedrich U., Zarin T., Fritsch A.W., Mittasch M., Domingues A., Hersemann L., Jahnel M., Richter D., Guenther U.P., Hentze M.W., Moses A.M., Hyman A.A. (2020). Condensation of Ded1p promotes a translational switch from housekeeping to stress protein production. Cell.

[35] Cruciat C.M., Dolde C., de Groot R.E., Ohkawara B., Reinhard C., Korswagen H.C., Niehrs C. (2013). RNA helicase DDX3 is a regulatory subunit of casein kinase 1 in Wnt-beta-catenin signaling. Science.

[36] Epling L.B., Grace C.R., Lowe B.R., Partridge J.F., Enemark E.J. (2015). Cancer-associated mutants of RNA helicase DDX3X are defective in RNA-stimulated ATP hydrolysis. J. Mol. Biol..

[37] Floor S.N., Condon K.J., Sharma D., Jankowsky E., Doudna J.A. (2016). Autoinhibitory interdomain interactions and subfamily-specific extensions redefine the catalytic core of the human DEAD-box protein DDX3. J. Biol. Chem..

[38] Valentin-Vega Y.A., Wang Y.D., Parker M., Patmore D.M., Kanagaraj A., Moore J., Rusch M., Finkelstein D., Ellison D.W., Gilbertson R.J., Zhang J., Kim H.J., Taylor J.P. (2016). Cancer-associated DDX3X mutations drive stress granule assembly and impair global translation. Sci. Rep..

[39] Oh S., Flynn R.A., Floor S.N., Purzner J., Martin L., Do B.T., Schubert S., Vaka D., Morrissy S., Li Y., Kool M., Hovestadt V., Jones D.T., Northcott P.A., Risch T. (2016). Medulloblastoma-associated DDX3 variant selectively alters the translational response to stress. Oncotarget.

[40] Sharma D., Putnam A.A., Jankowsky E. (2017). Biochemical differences and similarities between the DEAD-box helicase orthologs DDX3X and Ded1p. J. Mol. Biol..

[41] Beckham C., Hilliker A., Cziko A.M., Noueiry A., Ramaswami M., Parker R. (2008). The DEAD-box RNA helicase Ded1p affects and accumulates in Saccharomyces cerevisiae P-bodies. Mol. Biol. Cell.

[42] Mnaimneh S., Davierwala A.P., Haynes J., Moffat J., Peng W.T., Zhang W., Yang X., Pootoolal J., Chua G., Lopez A., Trochesset M., Morse D., Krogan N.J., Hiley S.L., Li Z. (2004). Exploration of essential gene functions via titratable promoter alleles. Cell.

[43] Alcazar-Roman A.R., Bolger T.A., Wente S.R. (2010). Control of mRNA export and translation termination by inositol hexakisphosphate requires specific interaction with Gle1. J. Biol. Chem..

[44] Iost I., Dreyfus M., Linder P. (1999). Ded1p, a DEAD-box protein required for translation initiation in Saccharomyces cerevisiae, is an RNA helicase. J. Biol. Chem..

[45] Robichaud N., Sonenberg N., Ruggero D., Schneider R.J. (2019). Translational control in cancer. Cold Spring Harb. Perspect. Biol..

[46] Gao X., Jiang L., Gong Y., Chen X., Ying M., Zhu H., He Q., Yang B., Cao J. (2019). Stress granule: A promising target for cancer treatment. Br. J. Pharmacol..

[47] Robichaud N., Sonenberg N. (2017). Translational control and the cancer cell response to stress. Curr. Opin. Cell Biol..

[48] Ingolia N.T., Ghaemmaghami S., Newman J.R., Weissman J.S. (2009). Genome-wide analysis *in vivo* of translation with nucleotide resolution using ribosome profiling. Science.

[49] Gerashchenko M.V., Lobanov A.V., Gladyshev V.N. (2012). Genome-wide ribosome profiling reveals complex translational regulation in response to oxidative stress. Proc. Natl. Acad. Sci. U. S. A..

[50] Jiang L., Gu Z.H., Yan Z.X., Zhao X., Xie Y.Y., Zhang Z.G., Pan C.M., Hu Y., Cai C.P., Dong Y., Huang J.Y., Wang L., Shen Y., Meng G., Zhou J.F. (2015). Exome sequencing identifies somatic mutations of DDX3X in natural killer/T-cell lymphoma. Nat. Genet..

[51] Snijders Blok L., Madsen E., Juusola J., Gilissen C., Baralle D., Reijnders M.R., Venselaar H., Helsmoortel C., Cho M.T., Hoischen A., Vissers L.E., Koemans T.S., Wissink-Lindhout W., Eichler E.E., Romano C. (2015). Mutations in DDX3X are a common cause of unexplained intellectual disability with gender-specific effects on Wnt signaling. Am. J. Hum. Genet..

[52] Wang X., Posey J.E., Rosenfeld J.A., Bacino C.A., Scaglia F., Immken L., Harris J.M., Hickey S.E., Mosher T.M., Slavotinek A., Zhang J., Beuten J., Leduc M.S., He W., Vetrini F. (2018). Phenotypic expansion in DDX3X - a common cause of intellectual disability in females. Ann. Clin. Transl. Neurol..

[53] Lennox A.L., Hoye M.L., Jiang R., Johnson-Kerner B.L., Suit L.A., Venkataramanan S., Sheehan C.J., Alsina F.C., Fregeau B., Aldinger K.A., Moey C., Lobach I., Afenjar A., Babovic-Vuksanovic D., Bezieau S. (2020). Pathogenic DDX3X mutations impair RNA metabolism and neurogenesis during fetal cortical development. Neuron.

[54] Bolger T.A., Wente S.R. (2011). Gle1 is a multifunctional DEAD-box protein regulator that modulates Ded1 in translation initiation. J. Biol. Chem..

[55] Liu F., Putnam A., Jankowsky E. (2008). ATP hydrolysis is required for DEAD-box protein recycling but not for duplex unwinding. Proc. Natl. Acad. Sci. U. S. A..

