# Supplementary material for: Medulloblastoma-associated mutations in the DEAD-box RNA helicase DDX3X/DED1 cause specific defects in translation
Source: J Biol Chem. 2021 Jan 16;296:100296. doi: 10.1016/j.jbc.2021.100296 (PMC7949108; doi:10.1016/j.jbc.2021.100296)
Supplement: Supplemental Figures and Tables [file mmc1.pdf]

<u>SUPPORTING INFORMATION</u>

<u>Contents:</u>

Supplementary References

Supplementary Table S1: Summary of phenotypes of *ded1-mam* mutants

Supplementary Table S2: List of yeast strains used

Supplementary Table S3: List of plasmids used

Supplementary Figure S1: Effects of expression level for medulloblastoma-associated mutations in *DDX3X/DED1*

Supplementary Figure S2: Decreases in *DED1* levels do not recapitulate the effects of medulloblastoma-associated mutations in *DDX3X/DED1*.

Supplementary References:

1. Thomas, B. J., and Rothstein, R. (1989) The genetic control of direct-repeat recombination in Saccharomyces: the effect of rad52 and rad1 on mitotic recombination at GAL10, a transcriptionally regulated gene. *Genetics* **123**, 725-738

2. Bolger, T. A., and Wente, S. R. (2011) Gle1 is a multifunctional DEAD-box protein regulator that modulates Ded1 in translation initiation. *J Biol Chem* **286**, 39750-39759

3. Aryanpur, P. P., Renner, D. M., Rodela, E., Mittelmeier, T. M., Byrd, A., and Bolger, T. A. (2019) The DEAD-box RNA helicase Ded1 has a role in the translational response to TORC1 inhibition. *Mol Biol Cell* **30**, 2171-2184

4. Mnaimneh, S., Davierwala, A. P., Haynes, J., Moffat, J., Peng, W. T., Zhang, W., Yang, X., Pootoolal, J., Chua, G., Lopez, A., Trochesset, M., Morse, D., Krogan, N. J., Hiley, S. L., Li, Z., Morris, Q., Grigull, J., Mitsakakis, N., Roberts, C. J., Greenblatt, J. F., Boone, C., Kaiser, C. A., Andrews, B. J., and Hughes, T. R. (2004) Exploration of essential gene functions via titratable promoter alleles. *Cell* **118**, 31-44

5. Sikorski, R. S., and Hieter, P. (1989) A system of shuttle vectors and yeast host strains designed for efficient manipulation of DNA in Saccharomyces cerevisiae. *Genetics* **122**, 19-27

6. Christianson, T. W., Sikorski, R. S., Dante, M., Shero, J. H., and Hieter, P. (1992) Multifunctional yeast high-copy-number shuttle vectors. *Gene* **110**, 119-122

7. Hilliker, A., Gao, Z., Jankowsky, E., and Parker, R. (2011) The DEAD-Box Protein Ded1 Modulates Translation by the Formation and Resolution of an eIF4F-mRNA Complex. *Mol Cell* **43**, 962-972

8. Chuang, R. Y., Weaver, P. L., Liu, Z., and Chang, T. H. (1997) Requirement of the DEAD-Box protein Ded1p for messenger RNA translation. *Science* **275**, 1468-1471

9. Sen, N. D., Zhou, F., Ingolia, N. T., and Hinnebusch, A. G. (2015) Genome-wide analysis of translational efficiency reveals distinct but overlapping functions of yeast DEAD-box RNA helicases Ded1 and eIF4A. *Genome Res* **25**, 1196-1205

10. Buchan, J. R., Muhlrad, D., and Parker, R. (2008) P bodies promote stress granule assembly in Saccharomyces cerevisiae. *J Cell Biol* **183**, 441-455

	Growth	Polysome M/P ratio	5'UTR reporter	Stress granules	ATPase activity	RNA affinity
WT	++++	0.8	21.4%	2.5%	1.00	13.0
T166A	+++	2.7	4.4%	4.2%	0.54	30.5
V168L	+	16.3		10.9%		
G189V	++++	1.0		7.1%		
T234M	+++	1.0	6.9%		0.24	
R235K	+	9.5				
R285C	++	2.8	1.8%	14.1%	0.60	7.1
R310W	++	2.6	1.0%	15.6%	1.74	10.9
L312F	+++	1.6	3.1%	8.4%		
F316S	++	1.3	3.0%	6.3%	1.20	10.3
M339I	++	2.9	1.6%		0.76	110.0
V364L	+++	1.1	9.0%	8.0%	1.04	11.2
S371F	++	1.3	4.8%	15.4%	0.14	11.0
R486H	+++		3.7%			
G488A	++++		24.6%		0.15	77.6
G494R	+++	2.8	7.3%	24.1%	0.83	14.6
P526L	+++	0.7	9.0%	37.2%	0.66	

Supplementary Table S1: Summary of phenotypes of *ded1-mam* mutants

Supplementary Table S2: Yeast strains used in this study

Strain Name	Genotype	Source
W303	*MATα ade2-1 ura3-1 his3-11,15 leu2-3,112 trp1-1 can1-100*	(1)
SWY4093	*MATα ded1::KANr ade2-1 ura3-1 his3-11,15 leu2-3,112 trp1-1 can1-100 +pCEN/URA3/DED1*	(2)
TBY52	*MATα ded1::KAN ade2-1 ura3-1 his3-11,15 leu2-3,112 trp1-1 can1-100 +pCEN/LEU2/DED1* (pSW3619)	(3)
TET_{off}-DED1	*MATa prDED1::KANr-tet07-TATA URA3::CMV-tTA his3-1 leu2-Δ0 met15-Δ0*	(4)
ded1-mam list (*MATα ded1::KAN ade2-1 ura3-1 his3-11,15 leu2-3,112 trp1-1 can1-100 +pCEN/LEU2/ded1-mam*):		
TBY53	*-R285C* (pTB38)	This study
TBY54	*-F316S* (pTB39)	This study
TBY55	*-P526L* (pTB42)	This study
TBY56	*-R492H* (pTB43)	This study
TBY57	*-T234M* (pTB40)	This study
TBY58	*-R335C* (pTB41)	This study
TBY59	*-S371F* (pTB44)	This study
TBY60	*-A184P* (pTB45)	This study
TBY61	*-R235K* (pTB59)	This study
TBY62	*-D313H* (pTB52)	This study
TBY63	*-M329R* (pTB54)	This study
TBY64	*-L312F* (pTB48)	This study
TBY65	*-D288V* (pTB49)	This study
TBY66	*-G488V* (pTB50)	This study
TBY67	*-R433C* (pTB51)	This study
TBY68	*-T166A* (pTB55)	This study
TBY69	*-V168L* (pTB56)	This study
TBY70	*-R486H* (pTB67)	This study
TBY71	*-L393Δ* (pTB66)	This study
TBY72	*-G189V* (pTB57)	This study
TBY73	*-T193A* (pTB63)	This study
TBY74	*-G262V* (pTB60)	This study
TBY75	*-L286V* (pTB62)	This study
TBY76	*-M339I* (pTB71)	This study
TBY77	*-V364L* (pTB74)	This study
TBY78	*-T490M* (pTB68)	This study
TBY79	*-R310W* (pTB73)	This study
TBY80	*-G494R* (pTB65)	This study
TBY81	*-E236Q* (pTB64)	This study
TBY82	*-A352S* (pTB76)	This study
TBY83	*-V454M* (pTB78)	This study
TBY87	*-G462V* (pTB88)	This study
TBY88	*-D326V* (pTB89)	This study

Supplementary Table S3: Plasmids used in this study

Plasmid Name	Description	Source
pRS315	*CEN/LEU2*	(5)
pSW3619	*CEN/LEU2/DED1*	(2)
pTB38	*CEN/LEU2/ded1-R285C*	This study
pTB39	*CEN/LEU2/ded1-F316S*	This study
pTB40	*CEN/LEU2/ded1-T234M*	This study
pTB41	*CEN/LEU2/ded1-R335C*	This study
pTB42	*CEN/LEU2/ded1-P526L*	This study
pTB43	*CEN/LEU2/ded1-D464Y*	This study
pTB44	*CEN/LEU2/ded1-S371F*	This study
pTB45	*CEN/LEU2/ded1-A184P*	This study
pTB46	*CEN/LEU2/ded1-R492H*	This study
pTB47	*CEN/LEU2/ded1-G284E*	This study
pTB48	*CEN/LEU2/ded1-L312F*	This study
pTB49	*CEN/LEU2/ded1-D288V*	This study
pTB50	*CEN/LEU2/ded1-G488A*	This study
pTB51	*CEN/LEU2/ded1-R433C*	This study
pTB52	*CEN/LEU2/ded1-D313H*	This study
pTB53	*CEN/LEU2/ded1-G261V*	This study
pTB54	*CEN/LEU2/ded1-M329R*	This study
pTB55	*CEN/LEU2/ded1-T166A*	This study
pTB56	*CEN/LEU2/ded1-V168L*	This study
pTB57	*CEN/LEU2/ded1-G189V*	This study
pTB59	*CEN/LEU2/ded1-R235K*	This study
pTB60	*CEN/LEU2/ded1-G262V*	This study
pTB61	*CEN/LEU2/ded1-H485Y*	This study
pTB62	*CEN/LEU2/ded1-L286V*	This study
pTB63	*CEN/LEU2/ded1-T193A*	This study
pTB64	*CEN/LEU2/ded1-E236Q*	This study
pTB65	*CEN/LEU2/ded1-G494R*	This study
pTB66	*CEN/LEU2/ded1-L393Δ*	This study
pTB67	*CEN/LEU2/ded1-R486H*	This study
pTB68	*CEN/LEU2/ded1-T490M*	This study
pTB70	*CEN/LEU2/ded1-I374Δ*	This study
pTB71	*CEN/LEU2/ded1-M339I*	This study
pTB72	*CEN/LEU2/ded1-T343P*	This study
pTB73	*CEN/LEU2/ded1-R310W*	This study
pTB74	*CEN/LEU2/ded1-V364L*	This study
pTB76	*CEN/LEU2/ded1-A352S*	This study
pTB77	*CEN/LEU2/ded1-A459Δ*	This study
pTB78	*CEN/LEU2/ded1-V454M*	This study
pTB88	*CEN/LEU2/ded1-G462V*	This study
pTB89	*CEN/LEU2/ded1-D326V*	This study
pTB90	*CEN/LEU2/ded1-Q240H*	This study
pTB109	*CEN/LEU2/ded1-V176S*	This study
pRS423	*2μ/HIS3*	(6)
pRP2086	*2μ/HIS3/GAL1/10prom::DED1-6xHis-HA-ProtA*	(7)
pTB75	*2μ/HIS3/GAL1/10prom::ded1-F316S-6xHis-HA-ProtA*	This study
pTB82	*2μ/HIS3/GAL1/10prom::ded1-L312F-6xHis-HA-ProtA*	This study
pTB83	*2μ/HIS3/GAL1/10prom::ded1-R285C-6xHis-HA-ProtA*	This study

pTB85	*2μ/HIS3/GAL1/10prom::ded1-V168L-6xHis-HA-ProtA*	This study
pTB86	*2μ/HIS3/GAL1/10prom::ded1-R235K-6xHis-HA-ProtA*	This study
pTB94	*2μ/HIS3/GAL1/10prom::ded1-A184P-6xHis-HA-ProtA*	This study
pTB95	*2μ/HIS3/GAL1/10prom::ded1-R486H-6xHis-HA-ProtA*	This study
pTB101	*2μ/HIS3/GAL1/10prom::ded1-A352S-6xHis-HA-ProtA*	This study
pTB102	*2μ/HIS3/GAL1/10prom::ded1-G462V-6xHis-HA-ProtA*	This study
pTB103	*2μ/HIS3/GAL1/10prom::ded1-S371F-6xHis-HA-ProtA*	This study
pRS313	*CEN/HIS3*	(5)
DED1	*CEN/HIS3/DED1*	(8)
pRS426	*2μ/URA3*	(6)
pTB105	*2μ/URA3/DED1*	(3)
pTB106	*2μ/URA3/ded1-S371F*	This study
pTB107	*2μ/URA3/ded1-R235K*	This study
pTB108	*2μ/URA3/ded1-G262V*	This study
pFJZ342	*CEN/URA3/5'UTR-RPL41A+CAA(23)-LUC*	(9)
pFJZ623	*CEN/URA3/5'UTR-RPL41A+CAA(23)+distal-stem-loop(3.7)-LUC*	(9)
pRP1657	*CEN/URA3/PAB-GFP/EDC3-mCherry*	(10)
pSW3576	*pET28a-DED1*	(2)
pTB170	*pET28a-ded1-T166A*	This study
pTB171	*pET28a-ded1-R285C*	This study
pTB172	*pET28a-ded1-F316S*	This study
pTB173	*pET28a-ded1-R310W*	This study
pTB174	*pET28a-ded1-V364L*	This study
pTB175	*pET28a-ded1-S371F*	This study
pTB176	*pET28a-ded1-G494R*	This study
pTB177	*pET28a-ded1-G488A*	This study
pTB178	*pET28a-ded1-P526L*	This study
pTB191	*pET28a-ded1-T234M*	This study
pTB192	*pET28a-ded1-M339I*	This study

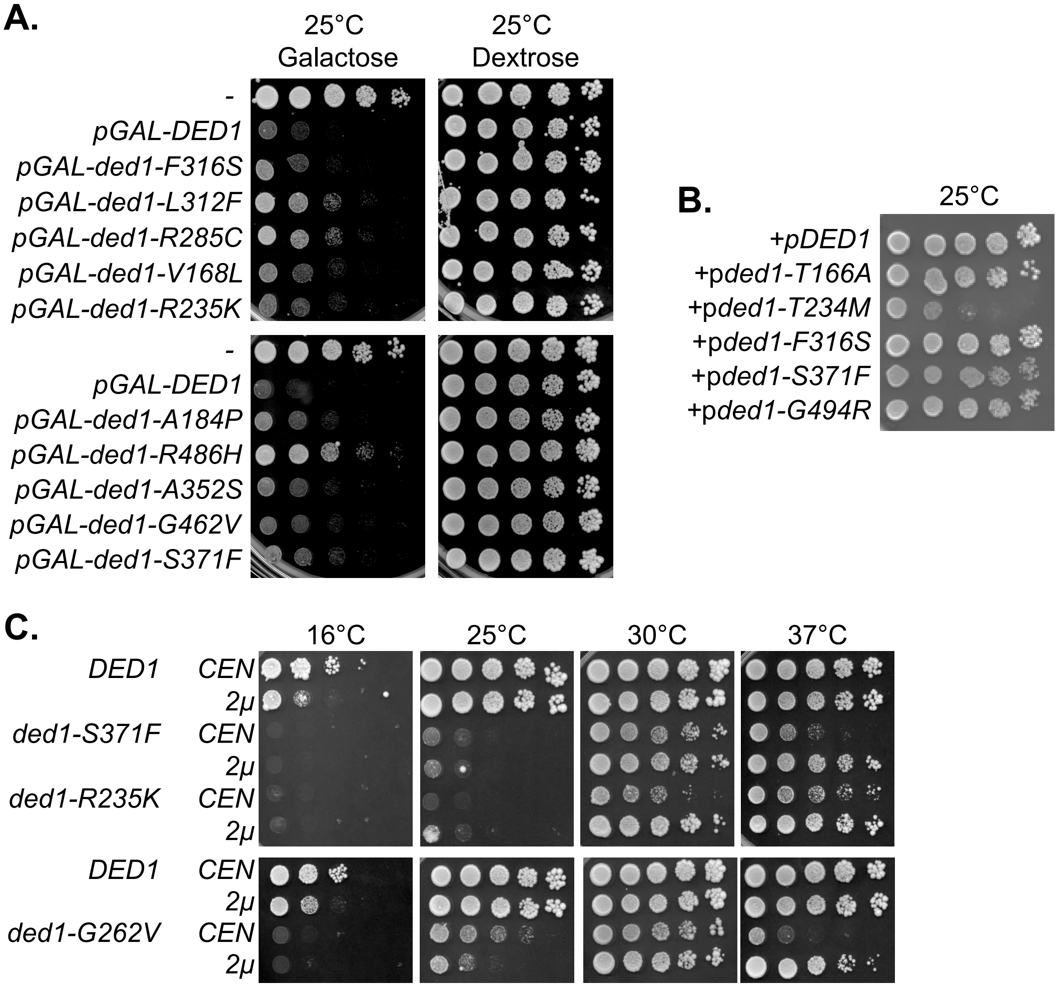

Supplementary Figure S1: Effects of expression level for medulloblastoma-associated mutations in *DDX3X/DED1*. (A) Wild-type cells containing high-copy plasmids with galactose-inducible *DED1* or the indicated *ded1-mam* mutants were grown on selective media containing galactose or dextrose. Representative 5x serial dilutions of cells are shown. Note the substantial effect on growth from wild-type *DED1* overexpression. (B) Wild-type cells containing low-copy plasmids expressing either wild-type *DED1* or *ded1-mam* mutants were grown at 25°C. Representative 5x serial dilutions of cells are shown. (C) Cells lacking endogenous *DED1* (*ded1-null*) but containing either low- (*CEN*) or high- (*2µ*) copy plasmids expressing wild-type *DED1* or the indicated *ded1-mam* mutants were grown on nutrient-rich agar at 16, 25, 30, and 37°C. Representative 5x serial dilutions of cells are shown.

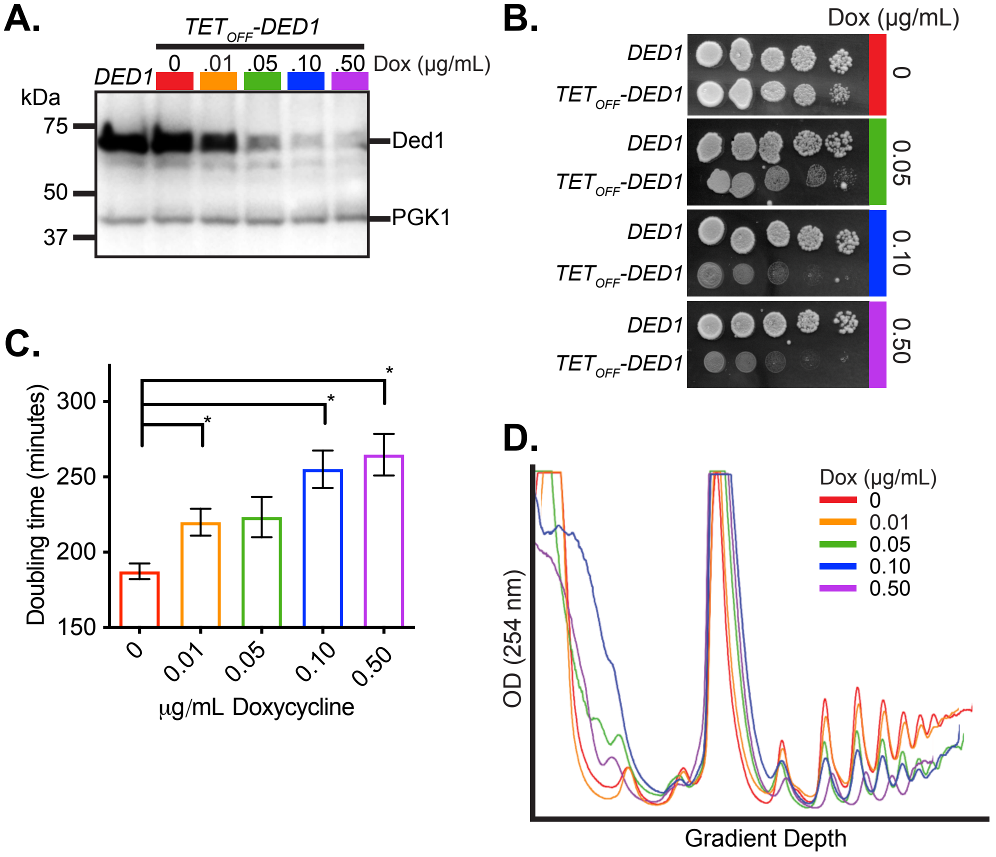

Supplementary Figure S2: Decreases in *DED1* levels do not recapitulate the effects of medulloblastoma-associated mutations in *DDX3X/DED1*. (A) Cells with a doxycycline-controlled *DED1* promoter (TET_{off}-*DED1*) were treated with 0, 0.01, 0.05, 0.10, and 0.50 µg/mL doxycycline for 8 hrs, and protein extracts were run on SDS-PAGE and blotted with anti-Ded1 and anti-PGK1 (loading control) antibodies. Wild-type *DED1* cells are also shown as a control for Ded1 expression level. (B) Wild-type and TET_{off}-*DED1* cells were grown on media containing doxycycline as in (A) at 30°C. Representative 5x serial dilutions of cells are shown. (C) TET_{off}-*DED1* cells were grown in media containing doxycycline as in (A) at 25°C, and culture growth was periodically measured by optical density. Doubling times are the mean and SEM from 3 independent trials. * $p < 0.05$ vs. no doxycycline. (D) Polyribosomal profiles were generated as in Figure 3 from TET_{off}-*DED1* cells treated with doxycycline as in (A), and representative profiles were overlaid to visualize progressive change in monosome and polysome peaks.
